# 967. Interprofessional Education of Medical Students in a Comprehensive HIV Care Coordination Elective

**DOI:** 10.1093/ofid/ofab466.1162

**Published:** 2021-12-04

**Authors:** Melanie Goebel, Natalia Rodriguez, Felicia Robinson, Shital Patel

**Affiliations:** Baylor College of Medicine, Houston, Texas

## Abstract

**Background:**

Interprofessional education (IPE) is critical in undergraduate medical student curriculum to promote teamwork, increase effective communication, and improve patient-centered care especially in medically underserved populations, including those living with HIV.

**Methods:**

Medical students participated in 2-week elective rotations at a freestanding, multidisciplinary HIV clinic providing comprehensive HIV care for more than 6,000 people in an urban, ethnically diverse, resource-limited population. The interprofessional faculty included physicians, pharmacists, case managers, social workers, service-linkage workers, substance use counselors, and medication access specialists. Students interviewed patients, rotated with at least four multidisciplinary health professionals at the clinic, and rounded with the HIV inpatient consult team and service linkage worker in the hospital. Each student completed a reflection paper on the barriers and facilitators of HIV care engagement. Student feedback was collected through course evaluations and debriefing sessions with course directors. Knowledge and ability to perform HIV-related services were assessed through student assessments at baseline and within 2 weeks of completing the rotation.

IPE competencies

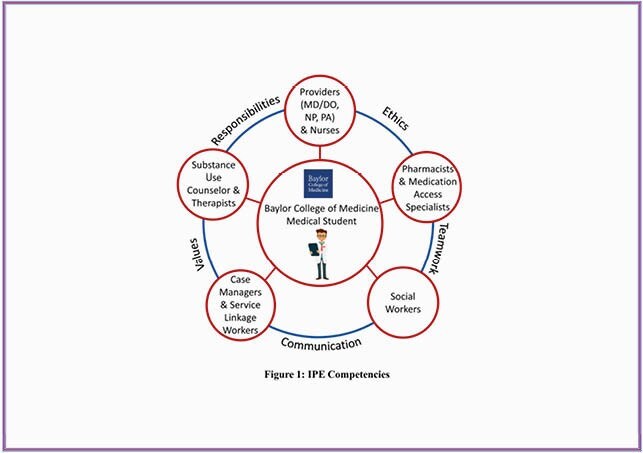

**Results:**

From January 20, 2020 to May 21, 2021, 17 medical students completed the 2-week elective (5 second-year students, 10 third-years, and 2 fourth-years). Student reflection papers demonstrated understanding of barriers to care, social determinants of health, and potential solutions to improve health outcomes. Open-ended feedback was positive, with many commenting on the benefits of learning from diverse healthcare professionals in HIV care. At follow up, 80% of students rated their knowledge of ideal functioning of interprofessional teams as very good or excellent. Students reported increased ability to deliver team-based care, provide services to culturally diverse people, and coordinate care for non-medical needs.

**Conclusion:**

Interprofessional education enhanced students’ knowledge of care coordination, interprofessional communication skills, competency in teamwork, and understanding of socioeconomic barriers to care in an underserved population with HIV.

**Disclosures:**

**All Authors**: No reported disclosures

